# Comparative Study of Heart Rate Variability Between Holstein Cattle and Mini Cows

**DOI:** 10.3390/ani16121909

**Published:** 2026-06-19

**Authors:** Carlos Javier Lainez Reyes, Simone Biagio Chiacchio, Paola Alejandra Montenegro Cuellar, Lucas Vinícius de Oliveira Ferreira, Dario Alejandro Cedeño Quevedo, Miriam Harumi Tsunemi, Renata Benedetti Cepinho, Rodrigo Francisco, Maria Lúcia Gomes Lourenço

**Affiliations:** 1 Department of Veterinary Clinics, School of Veterinary Medicine and Animal Science, São Paulo State University (UNESP), Botucatu 18618-970, SP, Brazil; c.reyes@unesp.br (C.J.L.R.); chiacchios@fmvz.unesp.br (S.B.C.); paola.cuellar@unesp.br (P.A.M.C.); lv.ferreira@unesp.br (L.V.d.O.F.); m.tsunemi@unesp.br (M.H.T.); renata.cepinho@unesp.br (R.B.C.); rodrigo.francisco@unesp.br (R.F.); 2 Department of Animal Health, Faculty of Animal Sciences, University of Nariño (UDENAR), Pasto 52001, Colombia; dacedenoq@gmail.com

**Keywords:** age effects, animal welfare, autonomic nervous system, heart rate variability, Holstein cows, miniature cattle, non-invasive monitoring, precision livestock farming

## Abstract

Heart rate variability (HRV) is an established biomarker of autonomic nervous system activity, yet its profile in miniature cattle remains poorly understood despite their growing importance in sustainable farming. This study compared HRV parameters between miniature and Holstein cows and assessed the influence of age on these profiles. Eighty clinically healthy female cattle (40 miniature, 40 Holstein), aged 2 to 8 years, were evaluated under field conditions using a Polar H10 heart rate monitor. RR intervals were analyzed using Kubios HRV software to obtain time- and frequency-domain indices. Miniature cows exhibited significantly lower heart rates and higher time-domain measures (RMSSD and SDNN) compared to Holsteins, while frequency-domain analysis revealed significant differences in LF, HF, and LF/HF ratio, suggesting group-associated differences in proportional autonomic balance. Age-stratified analysis revealed that these physiological distinctions were more pronounced in older cows (6–8 years). However, given the observational cross-sectional design of this study, confounding factors—specifically the different farm environments, management systems, and the active lactation status of the Holstein group—preclude attributing these differences solely to breed or body size. Therefore, these results suggest an associative physiological pattern rather than a definitive autonomic adaptation. Despite these limitations, portable HRV monitoring proved feasible under farm conditions, providing valuable preliminary baseline data that can inform future controlled studies on bovine cardiovascular welfare.

## Simple Summary

Miniature cattle are becoming popular as a sustainable alternative to traditional large dairy breeds, but very little is known about their heart health. In this study, we compared the heart rhythm patterns of miniature cows and Holstein cows to see if their smaller body size affects the way their hearts are regulated. We measured heartbeats using a non-invasive sensor placed on the animals and analyzed the natural variations that occur between beats, which reflect how the body balances stress and relaxation. Our results showed that miniature cows had slower heart rates and stronger natural variation between beats compared to Holstein cows, with differences becoming more noticeable in mature animals. However, because the cows were evaluated on different farms, and only the Holstein cows were actively producing milk, these findings represent preliminary associations rather than direct proof of breed-specific adaptations. Overall, this observational study shows that portable sensors are practical tools for field monitoring and provides a useful baseline for future, more controlled research on the cardiovascular well-being of miniature cattle.

## 1. Introduction

The global livestock sector is currently undergoing a transformation driven by the need to balance increasing food demand with environmental sustainability and resource optimization [1,2]. In this context, interest in miniature cattle breeds has grown considerably, as they are valued for their suitability for small properties, ease of handling, and potential for resource optimization in sustainable family farming models [3]. However, a significant gap remains in the scientific understanding of their cardiovascular physiology and how their unique body size influences their health and welfare.

Monitoring vital signs is fundamental for preventive veterinary medicine and animal welfare [4,5]. Physiological parameters such as heart rate and heart rate variability (HRV) help identify clinical problems and evaluate animal performance [6,7]. HRV refers to the variation in time between two consecutive heartbeats and serves as an indicator of the neurocardiac system’s function [6,7]. Generally, high HRV is associated with a healthy condition, while low HRV is linked to pathological conditions or stress [7,8,9,10,11].

Various studies have demonstrated the applicability of HRV as a tool for evaluating stress and welfare in different production species [6,12,13]. Nonetheless, miniature cows represent a particular physiological case where traditional allometric rules—suggesting higher heart rates in smaller animals—could be altered by breed-specific adaptations [5]. For these evaluations, it is crucial to use validated technology. Consequently, heart rate and RR intervals were recorded using a Polar H10^®^ sensor (Polar Electro Oy, Kempele, Finland). The device was selected for its high sampling frequency of 1000 Hz, which allows for millisecond-level precision in RR interval detection, a requirement for robust HRV analysis. The sensor was positioned on the left thoracic region, just caudal to the olecranon, ensuring optimal contact with the skin using conductive gel and secured by a specialized elastic thoracic strap adapted for large animals [14,15,16,17].

The main objective of this study was to compare heart rate variability parameters between miniature cows and Holstein cows under field conditions and to assess whether age categories were associated with differences in these profiles. We hypothesized that the two groups would exhibit distinct baseline HRV patterns; however, given the observational cross-sectional design and the differences in farm environment, management system, and lactation status, these differences were interpreted as associative physiological patterns rather than as direct evidence of breed- or body size-specific autonomic adaptation.

## 2. Materials and Methods

### 2.1. Ethical Approval

The study protocol was reviewed and approved by the Animal Use Ethics Committee (CEUA) of the School of Veterinary Medicine and Animal Science (FMVZ) at UNESP, Botucatu Campus, under protocol number 000.294. All procedures were conducted in compliance with applicable standards and Brazilian regulations for animal experimentation.

### 2.2. Animals and Study Design

A cross-sectional study was conducted involving 80 female cattle to compare cardiac autonomic profiles. The animals were assigned to two distinct groups based on breed and body conformation: Group 1 (G1) comprised 40 miniature cows sourced from Fazenda São Benedito (located in Avaré and Itatinga, SP), and Group 2 (G2) comprised 40 conventional Holstein cows from Fazenda Moura (located in Botucatu, SP). Inclusion criteria required animals to be aged between 2 and 8 years, clinically healthy upon veterinary examination, and non-pregnant. The Holstein cows (G2) had a mean Body Condition Score (BCS) of 3.0 on a 5-point scale and were in active lactation at the time of evaluation. The miniature cows (G1) were non-lactating and exhibited BCS consistent with breed maintenance standards. All animals were fully adapted to human handling and routine farm procedures. Animals with historical or clinical signs of systemic, metabolic, or cardiovascular disease, as well as those undergoing pharmacological treatments affecting the autonomic nervous system, were excluded.

**Figure 1:**
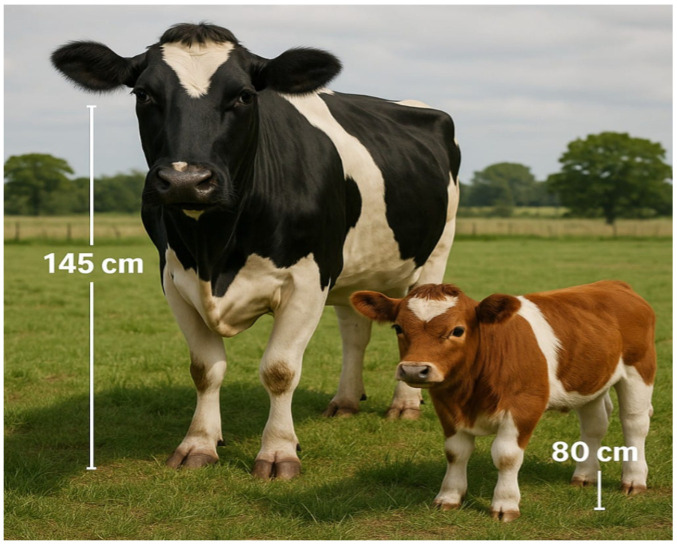
Size comparison between a high-stature cow (Holstein) and a miniature cow. Measurements reflect height at the withers (Source: generated with DALL-E 3, OpenAI, San Francisco, CA, USA, 2025).

Because the two groups were recruited from different farms and production systems, breed or body conformation was inherently confounded with farm-level factors, including environmental conditions, nutritional management, housing facilities, and routine handling practices. In addition, the Holstein cows were in active lactation, whereas the miniature cows were non-lactating. Therefore, the study was designed and interpreted as an observational cross-sectional comparison, and the individual contributions of breed, body size, management system, and lactation status could not be statistically isolated (Figure 1).

### 2.3. Data Collection and Equipment

**Figure 2:**
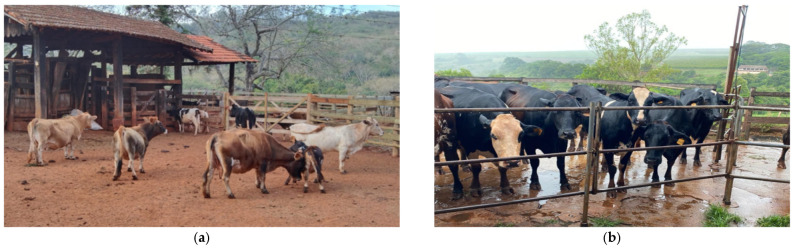
Animals in the handling corral, at rest, with access to water and feed ad libitum after being led from the pasture: (**a**) miniature cows; (**b**) high-stature cows.

Measurements were performed in the morning following the animals’ first daily meal, maintaining a standardized sequence of events across both properties. The animals were gently led from the pasture to a quiet handling corral, where they rested with access to water and feed ad libitum for approximately 30 min to minimize external and transport-induced stressors (Figure 2).

**Figure 3:**
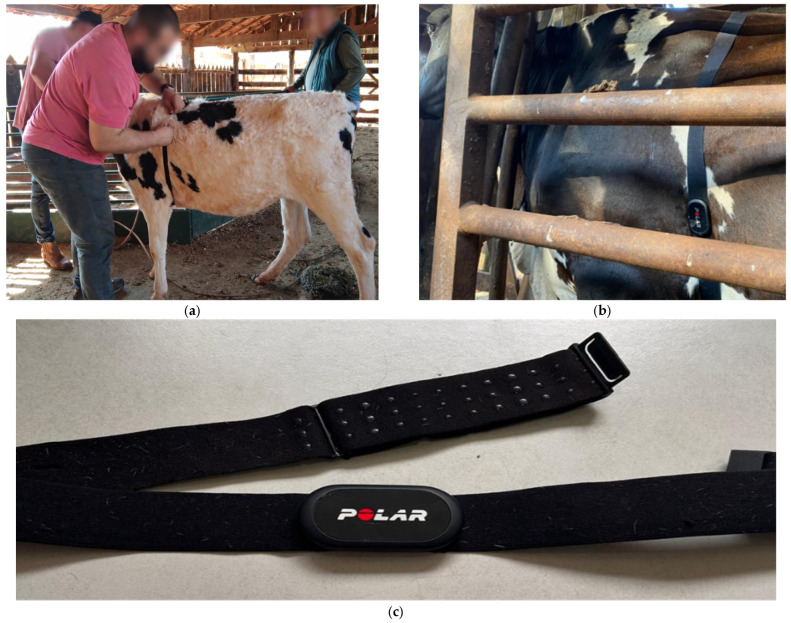
(**a**) Procedure for positioning the Polar H10 heart rate sensor on a miniature cow; (**b**) positioning of the sensor in the thoracic region of a normal-sized cow with the animal properly secured in a suitable squeeze chute; (**c**) Polar H10 heart rate sensor and its corresponding elastic chest strap.

Continuous cardiac monitoring was performed for 10 min per animal using a non-invasive portable Polar H10^®^ heart rate monitor (Polar Electro Oy, Kempele, Finland). To ensure high signal quality, a further 15 min stabilization and equipment adaptation period was provided after fitting the device. The sensor was fixed using an adjustable elastic chest strap positioned behind the forelimbs at the thoracic level, with the electrodes adjusted near the scapula and elbow joint. Conductive gel or water was applied to the skin to ensure optimal electrical conductivity without the need for trichotomy. Data were transmitted via to the Elite HRV smartphone application (Elite HRV LLC, Asheville, NC, USA) with a high-resolution sampling frequency of 1000 Hz, capturing exact RR intervals for subsequent variability analysis (Figure 3).

### 2.4. Signal Processing and HRV Analysis

The recorded RR interval series were exported in text format (.txt) and analyzed using Kubios HRV Scientific software (version 4.3, Kubios Oy, Kuopio, Finland). To guarantee the reliability of the cardiac autonomic metrics, each tachogram was visually inspected for technical anomalies or noise. Ectopic beats and short-term artifacts were automatically detected and corrected using the software’s standardized medium threshold filter. RR interval series displaying an artifact rate higher than 5% were excluded from final statistical processing to ensure data robustness.

**Figure 4:**
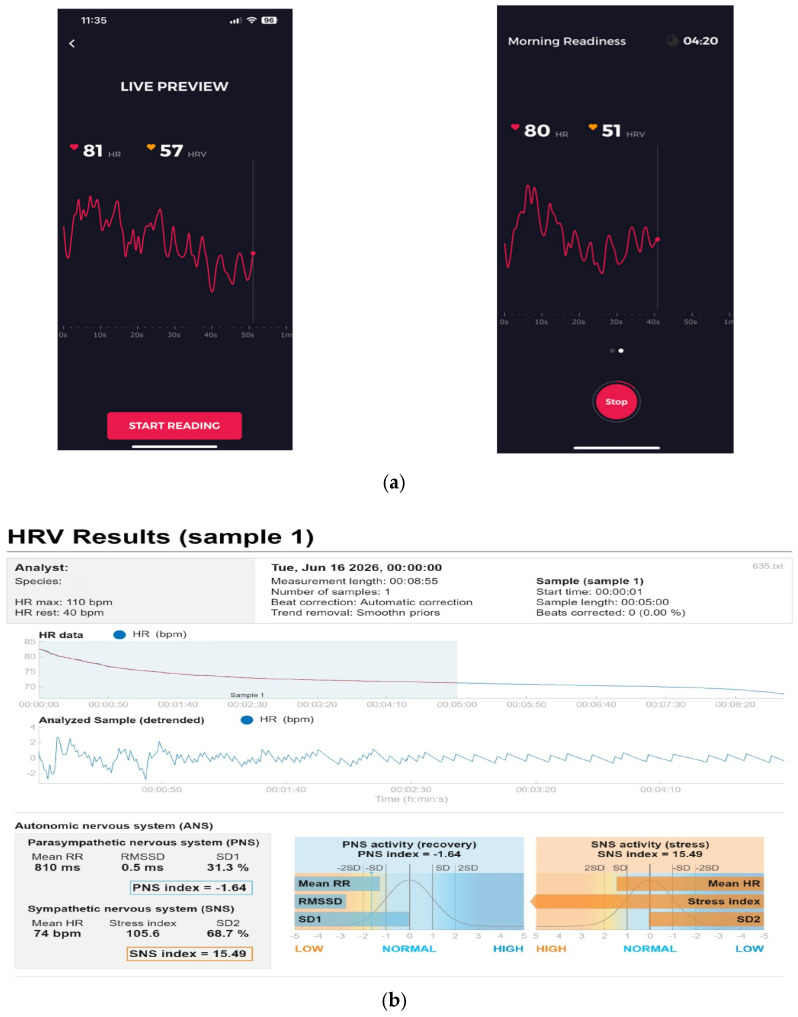
(**a**) Elite HRV application used for monitoring and recording RR intervals via Bluetooth; (**b**) interface of the Kubios HRV Scientific software used for analyzing heart rate variability (HRV) autonomic parameters.

The extracted time-domain indices included the mean heart rate (HR, bpm), mean RR interval (ms), standard deviation of normal-to-normal intervals (SDNN, ms), and the root mean square of successive differences (RMSSD, ms). Frequency-domain parameters were calculated using the Fast Fourier Transform (FFT) method, extracting low-frequency power (LF: 0.04–0.15 Hz), high-frequency power (HF: 0.15–0.40 Hz), and the LF/HF ratio as an index of sympathovagal balance. Global autonomic balance was further described using the software’s Sympathetic Nervous System (SNS) and Parasympathetic Nervous System (PNS) indices (Figure 4).

### 2.5. Statistical Analysis

Data normality was verified using the Shapiro–Wilk test. Variables with a normal distribution were compared between G1 and G2 using Student’s *t*-test, while non-normally distributed data were compared using the Mann–Whitney U test. Significance was set at *p* < 0.05. To evaluate autonomic behavior across development, data were stratified into two age categories: 2–5 years and 6–8 years. Statistical analyses were performed using Microsoft Excel (Microsoft Corporation, Redmond, WA, USA) and R software (version 4.3.1, R Foundation for Statistical Computing, Vienna, Austria). Because multiple HRV parameters were analyzed simultaneously, the results were interpreted with caution to minimize the risk of Type I error inflation. Given the exploratory nature of this physiological study and the interdependence among HRV variables, emphasis was placed on consistency of biological patterns across related autonomic indices rather than on isolated *p*-values alone.

## 3. Results

### 3.1. Heart Rate and Time-Domain HRV Parameters

From the initial 80 animals evaluated, two RR interval series (one from G1 and one from G2) presented an artifact rate higher than 5% and were therefore excluded from the final statistical processing, as established in the methodology. Consequently, the final analyzed dataset comprised 78 animals (*n* = 39 for G1; *n* = 39 for G2). The comparative analysis identified statistically significant differences in selected HRV parameters between the two groups. Miniature cows (G1) exhibited a significantly lower median heart rate (64.0 bpm; IQR: 14.0) compared to conventional Holstein cows (G2), which recorded a median of 79.0 bpm (IQR: 10.0; *p* < 0.001). Consequently, the cardiac cycle duration, represented by the RR intervals, was significantly longer in G1 than in G2 (*p* < 0.001), reflecting the expected physiological inverse relationship between these metrics (Table 1).

In the time domain, miniature cattle demonstrated a profile characterized by higher overall variability. The RMSSD values were significantly superior in G1 compared to G2 (*p* = 0.003), suggesting greater short-term parasympathetic-related variability in G1 under the conditions evaluated. A similar pattern was observed for SDNN, which was significantly higher in G1 (*p* = 0.034), confirming greater total autonomic dispersion. Conversely, the pNN50 index remained exceptionally low in both groups and did not present a statistically significant difference (*p* = 0.176), suggesting a lower sensitivity of this specific index for baseline bovine assessments.

### 3.2. Frequency-Domain Parameters and Global Autonomic Indices

Spectral analysis identified significant shifts in autonomic balance between the groups. In the frequency domain, miniature cows presented higher normalized low-frequency power (LF) compared to Holsteins (*p* = 0.012). Conversely, the high-frequency power (HF), reflecting absolute vagal tone, was significantly higher in the G2 group (*p* = 0.007). As a direct consequence of this distribution, the LF/HF ratio was significantly elevated in G1 (median: 1.65) relative to G2 (median: 1.47; *p* = 0.006), showing a relative sympathetic predominance in the miniature group when evaluated through proportional metrics.

**Figure 5:**
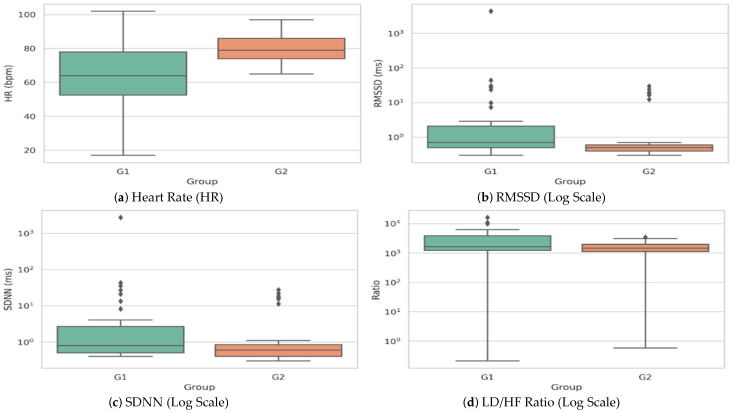
Comparative boxplots of key heart rate variability (HRV) parameters between miniature cows (G1) (green boxes) and Holstein cows (G2) (orange boxes): (**a**) heart rate (HR, bpm); (**b**) root mean square of successive differences (RMSSD, ms) displayed on a log scale; (**c**) standard deviation of normal-to-normal intervals (SDNN, ms) displayed on a log scale; (**d**) low-frequency-to-high-frequency (LF/HF) ratio displayed on a log scale. Boxes represent the interquartile range (IQR), horizontal lines indicate medians, and whiskers extend to 1.5× IQR. Diamond symbols represent individual outliers beyond the whiskers.

Regarding global autonomic parameters, a distinct pattern emerged: Holstein cows exhibited significantly higher absolute values for both the Sympathetic Nervous System (SNS) index (*p* = 0.034) and the Parasympathetic Nervous System (PNS) index (*p* = 0.0001). This indicates that while miniature cows maintain a higher proportional sympathetic tone (LF/HF ratio), conventional dairy cows display a greater absolute magnitude of total autonomic modulation (SNS and PNS indices) (Figure 5).

### 3.3. Age-Stratified Analysis

Stratification by age categories suggested that differences between groups were more evident in older animals (Table 2). In the younger subgroup (2 to 5 years; *n* = 23 for G1, *n* = 9 for G2), significant differences between G1 and G2 were strictly restricted to heart rate (*p* = 0.037) and RR intervals (*p* = 0.011). Other parameters, including RMSSD (*p* = 0.940), SDNN (*p* = 0.905), and the LF/HF ratio (*p* = 0.967), did not differ statistically in younger animals, suggesting a more homogeneous baseline autonomic tone during early development.

In contrast, within the older subgroup (6 to 8 years; *n* = 16 for G1, *n* = 30 for G2), physiological divergence became widespread and highly pronounced. Older miniature cows (G1) maintained a remarkably lower heart rate (median: 59.80 bpm) than their Holstein counterparts (median: 79.13 bpm; *p* < 0.0001). Furthermore, the proportional sympathetic dominance in older miniature cows was confirmed by a significantly lower HF component (*p* = 0.007) and a markedly superior LF/HF ratio (*p* = 0.006), which reached a median of 3.91 compared to 1.59 in G2. This maturation effect is further substantiated by the absolute indices, where G2 consistently retained higher SNS (*p* = 0.034) and PNS (*p* = 0.0001) absolute magnitudes.

## 4. Discussion

The present study aimed to compare heart rate variability (HRV) parameters between Holstein cattle and miniature cows and to describe group-associated patterns in autonomic cardiac regulation under field conditions. Contrary to traditional allometric expectations—which generally associate a smaller body mass with higher resting heart rates—our results demonstrated that miniature cows (G1) presented a significantly lower basal heart rate and greater overall time-domain variability (RMSSD and SDNN) compared to Holstein cows (G2).

Biologically, the lower heart rate and longer RR intervals observed in the miniature group suggest a distinct baseline autonomic profile that contradicts classic allometric scaling. Rather than representing a strictly size-related effect, this pattern may reflect the combined influence of breed-related characteristics, productive status, environmental conditions, and management practices. Furthermore, the wider data dispersion and higher interquartile ranges (IQRs) found in G1 for RMSSD and SDNN indicate a highly dynamic regulation of beat-to-beat adjustments under resting conditions. This wider variability in cardiac cycle duration may indicate greater heterogeneity in beat-to-beat regulation within G1, although its biological meaning should be interpreted cautiously due to the observational nature of the study.

In contrast to the initial whole-group interpretations, the frequency-domain indices provided a crucial, complementary perspective on the sympathovagal balance. While miniature cows displayed higher global time-domain oscillations, their significantly higher LF/HF ratio indicates a greater relative sympathetic predominance under resting conditions compared to Holsteins. This apparent paradox—coexisting high short-term temporal variability (RMSSD) with a higher proportional sympathetic tone (LF/HF)—can be reconciled when analyzing the absolute global metrics. Holstein cows (G2) exhibited significantly higher absolute values for both the SNS and PNS indices, demonstrating a greater absolute magnitude of total autonomic modulation. This indicates that conventional high-stature dairy cattle maintain a more robust absolute neurocardiac tone, whereas miniature cattle regulate homeostasis via a more sensitive and proportionally sympathetic-shifted balance.

A critical factor that must be considered when interpreting these autonomic differences is the environmental and management context of the study. The two experimental groups were sourced from separate properties in geographically distinct locations, meaning that breed-associated traits are fundamentally coupled with farm-level variables, including nutritional regimens, daily handling histories, microclimates, and facility designs. Autonomic cardiac regulation is highly sensitive to these external modulators [18,19,20,21,22,23]. Additionally, the Holstein group consisted of cows in active lactation, a productive stage well-documented to induce substantial metabolic demands, elevated basal heart rates, and alterations in sympathetic tone to support milk production. Conversely, the miniature cows evaluated were non-lactating. Therefore, the divergent cardiac patterns identified cannot be attributed solely to body size or genetics; instead, they represent a complex, integrated response reflecting the interaction between breed characteristics, productive status, and specific environmental management. Therefore, the present results should not be interpreted as demonstrating that breed or body size independently determines autonomic regulation. Instead, they indicate that the two evaluated groups differed in HRV profiles within their respective physiological and management contexts.

The stratification of results by age further substantiated the role of physiological maturity in shaping these cardiac profiles. The lack of widespread autonomic differences in the younger subgroup (2 to 5 years) suggests that distinct regulatory patterns develop progressively. In older animals (6 to 8 years), the divergence became highly pronounced, with miniature cows exhibiting an even lower heart rate and a marked increase in the LF/HF ratio. As animals mature, their autonomic nervous system refines its environmental homeostatic responses. This age-dependent accentuation underscores the necessity of establishing age-specific baseline references when utilizing HRV as a clinical or welfare indicator in livestock, as direct comparisons across different age brackets may lead to inaccurate physiological interpretations.

From a precision livestock perspective, the non-invasive monitoring of RR intervals using portable chest-strap sensors proved highly feasible and practical under field conditions, avoiding the constraints of restrictive clinical settings [24,25]. However, a clear limitation of the current study is its cross-sectional design, which captures only a momentary physiological snapshot and precludes the evaluation of circadian rhythms or seasonal variations. Furthermore, while portable telemetric systems like the Polar H10 have been validated against gold-standard electrocardiography (ECG) in conventional horses and calves, specific concurrent validation was not performed within our miniature cattle cohort. Future research should focus on longitudinal approaches that monitor the same individuals across different productive cycles and climatic seasons, validating the sensor output against a continuous portable ECG. Exploring the correlations between baseline HRV profiles, behavioral reactivity tests, and metabolic biomarkers will be essential to fully clarify whether these distinct autonomic patterns confer adaptive advantages or specific resilience to production stressors in modern livestock systems.

## 5. Conclusions

The present observational cross-sectional study identified differences in selected HRV parameters between miniature cattle and conventional Holstein cows under field conditions, with miniature cows showing lower resting heart rates and a proportionally higher sympathetic tone. However, as an observational cross-sectional comparison, these findings must be interpreted cautiously. The observed differences cannot be exclusively attributed to breed conformation or allometric scaling, as environmental management, nutritional factors, and the active lactation status of the Holstein group represent significant confounding variables. Consequently, our results highlight a preliminary physiological association rather than a definitive breed-specific autonomic adaptation. Despite these limitations, this study provides useful preliminary insights into the HRV characteristics of miniature cattle. The use of portable telemetric monitors was feasible under field conditions and may support future non-invasive assessments of cardiac autonomic activity in cattle. These baseline findings establish a solid foundation for future controlled longitudinal studies to better clarify whether the observed HRV patterns are related to breed, body size, age, lactation status, or management conditions.

## Figures and Tables

**Table 1:** General comparison of HRV parameters between miniature (G1) and Holstein (G2) cows.

Parameter	Group	Median	IQR	*p*-Value	Test Used
HR (bpm)	G1	64.0	14.0	<0.001	Mann-Whitney U
	G2	79.0	10.0		
RR (ms)	G1	934.0	405.0	<0.001	Mann-Whitney U
	G2	763.0	75.0		
RMSSD (ms)	G1	0.7	0.95	0.003	Mann-Whitney U
	G2	0.5	0.20		
SDNN (ms)	G1	0.8	1.10	0.034	Mann-Whitney U
	G2	0.6	0.25		
LF/HF	G1	1.65	2.80	0.006	Mann-Whitney U
	G2	1.47	0.76		

Data are presented as median and interquartile range (IQR). HR: heart rate; RR: interval between beats; RMSSD: root mean square of successive differences; SDNN: standard deviation of NN intervals; LF/HF: ratio between low- and high-frequency components.

**Table 2:** Comparison of HRV parameters by age group between G1 and G2.

Parameter	Age Range	Median G1	Median G2	*p*-Value
HR (bpm)	2 to 5 years (G1: *n* = 23, G2: *n* = 9)	70.91	82.11	0.037
	6 to 8 years (G1: *n* = 16, G2: *n* = 30)	59.80	79.13	<0.0001
RR (ms)	2 to 5 years (G1: *n* = 23, G2: *n* = 9)	899.57	743.00	0.011
	6 to 8 years (G1: *n* = 16, G2: *n* = 30)	1212.69	763.27	0.020
HF (nu)	2 to 5 years (G1: *n* = 23, G2: *n* = 9)	40.79	39.65	0.856
	6 to 8 years (G1: *n* = 16, G2: *n* = 30)	27.26	40.39	0.007
LF/HF	2 to 5 years (G1: *n* = 23, G2: *n* = 9)	2.44	1.61	0.324
	6 to 8 years (G1: *n* = 16, G2: *n* = 30)	3.91	1.59	0.006

Values are expressed as medians. HR: heart rate; RR: interval between beats; HF: high frequency; LF/HF: low-frequency/high-frequency ratio.

## Data Availability

The data presented in this study are available in the master’s dissertation deposited in the repository of the UNESP Botucatu Library.

## Abbreviations

The following abbreviations are used in this manuscript:

HRV	Heart Rate Variability
ANS	Autonomic Nervous System
RR	Interval R–R
SDNN	Standard Deviation of NN Intervals
RMSSD	Root Mean Square of Successive Differences
FFT	Fast Fourier Transform
LF	Low Frequency
HF	High Frequency
LF/HF	Low-Frequency/High-Frequency Ratio
CEUA	Comissão de Ética no Uso de Animais
UNESP	Universidade Estadual Paulista
